# How to Unravel the Key Functions of Cryptic Oomycete Elicitin Proteins and Their Role in Plant Disease

**DOI:** 10.3390/plants10061201

**Published:** 2021-06-12

**Authors:** Aayushree Kharel, Md Tohidul Islam, James Rookes, David Cahill

**Affiliations:** School of Life and Environmental Sciences, Deakin University, Geelong Waurn Ponds Campus, Waurn Ponds, VIC 3216, Australia; kharelaa@deakin.edu.au (A.K.); md.islam2@deakin.edu.au (M.T.I.); james.rookes@deakin.edu.au (J.R.)

**Keywords:** elicitins, oomycetes, *Phytophthora*, plant–pathogen interaction, virulence, avirulence, CRISPR/Cas, omics

## Abstract

Pathogens and plants are in a constant battle with one another, the result of which is either the restriction of pathogen growth via constitutive or induced plant defense responses or the pathogen colonization of plant cells and tissues that cause disease. Elicitins are a group of highly conserved proteins produced by certain oomycete species, and their sterol binding ability is recognized as an important feature in sterol–auxotrophic oomycetes. Elicitins also orchestrate other aspects of the interactions of oomycetes with their plant hosts. The function of elicitins as avirulence or virulence factors is controversial and is dependent on the host species, and despite several decades of research, the function of these proteins remains elusive. We summarize here our current understanding of elicitins as either defense-promoting or defense-suppressing agents and propose that more recent approaches such as the use of ‘omics’ and gene editing can be used to unravel the role of elicitins in host–pathogen interactions. A better understanding of the role of elicitins is required and deciphering their role in host–pathogen interactions will expand the strategies that can be adopted to improve disease resistance and reduce crop losses.

## 1. Introduction

Elicitins are proteins characterized as pathogen-associated molecular patterns (PAMPs) that are exuded by oomycete species and, mainly, pathogens in the genera *Phytophthora* and *Pythium* [1]. Identified in the late 1980s as small 10-kDa proteins [2], elicitins are composed of 98 amino acids, including six cysteine residues in conserved positions [3]. Elicitins were initially described as extracellular sterol-binding proteins that transport sterols from the plant plasma membrane to a pathogen [4], and this elicitin–sterol complex could trigger specific signaling responses in the plant host. However, in more recent years, there has been growing evidence [5] that sterol binding and the elicitation of plant defense responses are two separate and independent events.

Since the 1990s, the role of elicitins as avirulence factors has been postulated and based around two major discoveries: firstly, the concept and verification of PAMPs and, secondly, the induction of a hypersensitive reaction (HR) in *Nicotiana* leaves or suspension-cultured tobacco cells treated with an elicitin [6]. However, recent findings [1,5,7,8] have suggested the involvement of elicitins in enhancing pathogenicity in certain plant–pathogen interactions. Plants and pathogens are in an arms race; most of the time, plants prevail, but sometimes, the pathogen does, and disease results, and as many have suggested - for example, Dodds et al. [9] - an understanding of the key factors that support pathogenicity may provide significant opportunities for enhanced disease control. Similarly, Buscaill and van der Hoorn [10] outlined the central role of PAMPS in host–pathogen interactions and that a better understanding of these molecules will likely underpin the discovery of new techniques for disease prevention.

Here, we concentrate on the application of modern tools to elucidate the role of elicitins and decipher their underlying significance in plant–pathogen interactions. Recently, multi-omics approaches have led to breakthroughs in our understanding of plant disease [11,12], and the application of efficient genome-editing tools such as CRISPR/Cas (clustered regularly interspaced short palindromic repeats/CRISPR-associated protein) has substantially broadened the opportunity to dissect the complexities of plant–pathogen interactions [13,14,15,16,17]. We focus on the application of techniques that can now be applied to enable a full understanding of the function of elicitins.

## 2. Distribution of Elicitins within the Oomycetes 

Extensive genomic studies show that elicitins are secreted by many oomycetes but are absent in all non-oomycete microorganisms. Basic Local Alignment Search Tool (BLAST) searches of elicitin domain-containing proteins, signal sequences and core promoter sequences show elicitin homologs in a range of *Phytophthora, Pythium, Pseudoperonospora, Hyaloperonospora* and *Albugo* species. The elicitins described so far belong to the more closely related subgroups of oomycetes, in contrast to species from the Saprolegnales, a more distantly related oomycete lineage [18,19,20]. 

## 3. Cell Wall-Bound Elicitins and Elicitin-Like Proteins

**Figure 1:**
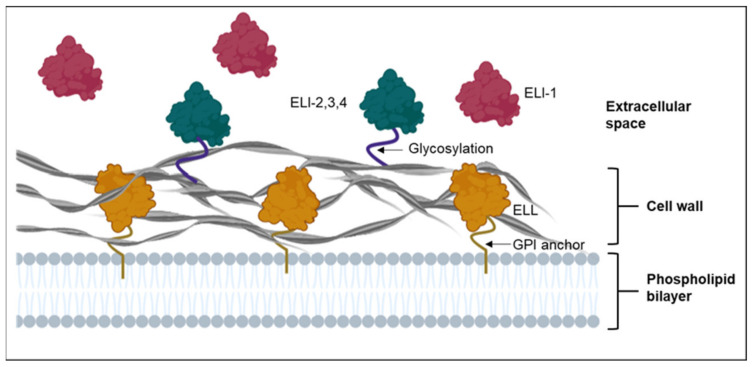
Characterization of elicitins and elicitin-like proteins based on their localization. Class I elicitins (ELI-1) (represented in red) such as cryptogein, which is produced by *Phytophthora cryptogea* [20], are released into the extracellular space. Class II, III and IV elicitins (ELI-2, -3 and -4) (represented in green), such as inf2A produced by *P. infestans* [23], are hypothesized to be linked to the cell wall by glycosylation of the C-terminal domain. Other elicitin-like (ELL) proteins (represented in orange) are postulated to be attached to the cell membrane with a glycosylphosphatidylinositol (GPI) anchor.

The phylogenetic analysis of elicitins [20,21] revealed seventeen clades: four were canonical elicitin (ELI) clades, and the remaining thirteen were elicitin-like (ELL) clades. The ELI-1 clade is the largest group of elicitins and are reported to be secreted into liquid culture. The ELI-2, 3 and 4 clades are hypothesized to be linked to the cell wall by extensive glycosylation of the C-terminal domain. On the other hand, ELL clades are hypothesized to be anchored to the cell membrane by glycosylphosphatidylinositol (GPI) anchors (Figure 1). Proteins associated with the cell wall and plasma membrane of oomycetes are vital for interacting with and sensing the local environment [21], but understanding their role has been challenging due to the difficulties in their isolation using conventional approaches. Nevertheless, an early investigation with *Phytophthora infestans* [22] identified two novel proteins (inf2A and inf2B) within ELI-2 that were later shown to act as avirulence factors that induced HR in *Nicotiana benthamiana* [23]. In addition, a more recent study by Islam et al. [24] that used a nano liquid chromatography-mass spectrometry (LC-MS/MS)-based approach to identify cell wall-bound elicitins and elicitin-like proteins of *Phytophthora cinnamomi*, found the major *P. cinnamomi* elicitin, beta-cinnamomin, to be cell wall-bound. Therefore, this sensitive and specific approach, along with others discussed further here, can now form a useful platform to explore the different types of elicitins and ELL proteins produced by other oomycete species.

## 4. Elicitins as Virulence or Avirulence Factors?

**Figure 2:**
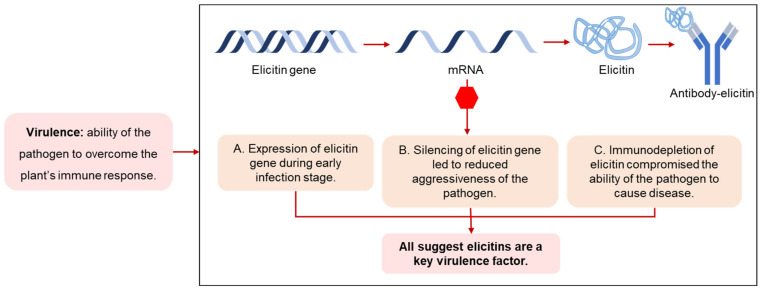
Compilation of three independent studies that support the role of elicitins as a virulence factor. **(A**) Expression of the *beta-cinnamomin* gene (elicitin of *P. cinnamomi*) during pathogen growth and lupin infection suggested a vital role of the protein in pathogen development and in the early stages of root infection [24]. (**B**) Inhibition of the expression of the *beta-cinnamomin* gene reduced the pathogenicity of *P. cinnamomi* in cork oak [27]. (**C**) *Fagus sylvatica* roots that were pre-incubated with an alpha-plurivorin antibody remained healthy in contrast to the plants that were not exposed to the antibody. Immunodepletion of alpha-plurivorin (elicitin of *Phytophthora plurivora*) compromised the penetration capacity of the pathogen [28].

**Figure 3:**
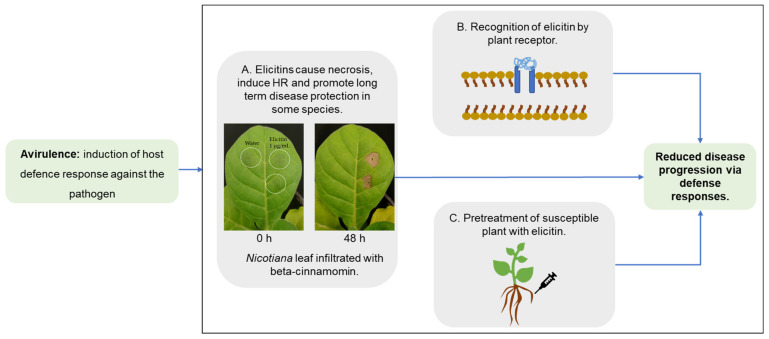
Summary of studies that support the role of elicitins as an avirulence factor. (**A**) The hallmark response of avirulence by the formation of a necrotic lesion in *Nicotiana* leaves upon infiltration with certain elicitins (such as cryptogein and beta-cinnamomin) and the induction of a HR [2] and unpublished results. (**B**) Transfer of the elicitin receptor into a cultivated potato resulted in an enhanced resistance to *P. infestans* [29]. (**C**) *Quercus suber* and *Quercus ilex* roots pretreated with alpha-cinnamomin (elicitin of *P. cinnamomi*) showed an overall reduction in the ability of the pathogen to colonize the roots in contrast to those roots not pretreated with alpha-cinnamomin [30].

As a result of the studies carried out to date, it has been established that elicitins may have opposing functions in plant–pathogen interactions: on the one hand, through the promotion of virulence via enabling the pathogen to cause disease (Figure 2) or on the other, avirulence, by the induction of plant defense responses (Figure 3). The contradictory role of these proteins in terms of pathogenicity is clear; for example, transcriptomic data has shown elicitin genes of *Phytophthora phaseoli* to be differentially upregulated in lima beans (*Phaseolus* spp.) during the infection stage, supporting a virulence role [25], whereas the induction of HR in *N. benthamiana* by elicitins that contain pathogen spread supports a role in avirulence [26]. 

In nature, the pathogen secretes elicitins, along with a complex mixture of other molecules (such as cell wall-degrading enzymes and effectors), which may lead to a different response in the plant when compared against the response to the purified form. For example, even though palmivorein, an elicitin of *Phytophthora palmivora*, induced a necrotic lesion in leaves of *Nicotiana* [31], the inoculation of *N. benthamiana* with the pathogen led to successful infection [32]. Thus, interpretation of the response of plants to purified elicitins must be undertaken with caution, as they may not fully reflect the actual interaction that occurs between an oomycete and its host. Hence, future research using the omics tools discussed in this review will help to reveal and define the responses of both the plant and the pathogen.

As per the zig-zag model [33], *Phytophthora-*plant interactions involve PTI–ETS–ETI, with an overlap between the various responses making it difficult to set boundaries between these events [34]. For example, based on our current understanding of the interactions in model plant–pathogen systems, Thomma et al. [34], along with Naveed et al. [35], concluded that a distinction between PAMPs and effectors cannot be strictly maintained. Thus, it has been proposed that moving beyond model pathosystems, and with the use of modern approaches to understand the molecular basis of plant–pathogen interactions, the dichotomy between elicitins and effectors will be elucidated. 

The differences in functions that elicitins may have, depending on the genotype of the host plant and other variables, have prompted at least one group to propose that the term ‘elicitins’ is redundant and introduced the concept of using the neutral and well-understood term ‘effector’ to also describe these proteins [27]. In contrast, Noman et al. [5] emphasized the uniqueness of the elicitin structure and sequence and rather identifies these proteins as a signature characteristic of oomycetes. Given that elicitins have been conserved in *Phytophthora* species over evolutionary time and their distinct structure and PTI response distinguishes them from other molecules, it is likely that elicitins will continue to be classed separately.

## 5. Application of Modern Tools and Techniques for Functional Analysis of Elicitins

In addition to improving breeding techniques, a more detailed and more tightly focused understanding of plant–pathogen interactions will help us to not only predict disease outbreaks but, also, to reduce crop losses. We have come a long way since the discovery of elicitins; however, ambiguity in the function of these proteins persists. Multiple studies have been conducted through in vitro experiments; however, an *in planta* infection with *Phytophthora* species, followed by monitoring the expression of relevant genes, including those that encode elicitins, will provide important insights. A microscopic technique, such as confocal microscopy coupled with the immunolabeling of elicitins and the use of transmission electron microscopy, has been shown to be important for the visualization of elicitins [24,36]. Furthermore, *in planta* study of elicitins has been limited, almost exclusively, to species of the *Nicotiana* genus. While these studies have been central to our current understanding of elicitins and their potential roles, we can now move on to focusing our studies on hosts for which whole genomes have been sequenced and annotated, such as those in the genera *Solanum*, *Eucalyptus* and *Glycine*, for which there are also well-known economic and environmental impacts caused by oomycetes. A combination of our current approaches, along with the newer techniques that are currently being used in other areas of plant science, can now be added to the toolbox that can be applied to plant–pathogen investigations in which elicitins are primarily involved.

### 5.1. Omics Approaches for Defining Functional Roles of Elicitins

**Figure 4:**
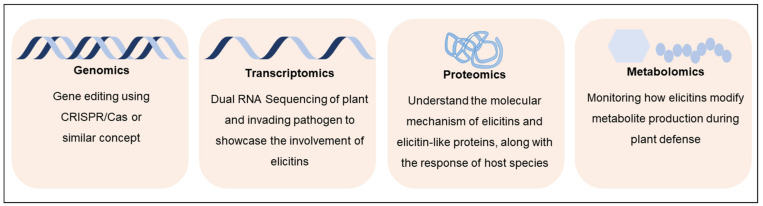
The four omics platforms that can be adapted to solve the underlying uncertainties around elicitin functions and to further understand their involvement in plant–pathogen interactions.

The integration of multiple omics technologies is a biological analysis approach that allows a better understanding of genotype–phenotype interactions upon the collection and combination of various data types, such as the genome, transcriptome, proteome, epigenome and metabolome. Omics has been identified as a mainstream approach to identify plant or pathogen targets for superior breeding techniques [37,38]. A combination of omics technologies (Figure 4), along with the currently used analytical chemistry techniques such as LC/MS, holds prospects for the enhancement of our understanding of elicitin-targeted host processes.

#### 5.1.1. Genomics and Next-Generation Sequencing

Advancements in next-generation sequencing (NGS) have facilitated the creation of genome libraries that can be used as a rich source of molecular markers that can allow us to better understand the pathogenicity, host preferences and effector proteins [39]. The whole-genome sequencing of pathogens has opened up pathways for an understanding of the evolutionary patterns and to identify specific pathogenicity genes and their annotations and functional characterizations. The alignment of various isolates of the same species to a single reference genome often leads to confusion, as we are left with multiple genes that do not align [40]. The recent introduction of the pangenome concept in the mid-2000s has assisted in addressing the variations that exists between different isolates of the same species, as it captures chromosomal variations and genomic plasticity, including differences in effector genes. We suggest, as others have [40,41], that, rather than limiting genomic studies based on the ‘reference genome paradigm’, the study of the pangenome should be adopted, as it better represents the genomic diversity of a species. More broadly, in regard to plant–pathogen interactions, to ensure successful invasions, pathogens possess accessory genes that constantly undergo sequence gains and losses [40]. Through pangenome studies, we can potentially unravel the extensive variations inherent in pathogen virulence factors and better understand the coevolution between a pathogen and their plant host.

#### 5.1.2. Transcriptomics

The use of the transcriptomic analysis tool RNA-sequencing (RNA-Seq) has proven to be a powerful method to identify infection- and disease-related genes in oomycete species. For example, following the generation of an RNA-Seq library by Reitmann et al. [42], the expression of elicitin genes produced by *P. cinnamomi* was analyzed over different life stages (mycelia, sporulating mycelia, zoospores and cysts and germinating cysts) and confirmed using RT-qPCR. They found, for example, that the expression of an elicitin-like gene (U4987) was maximal during zoospore development but slightly lower at the cyst and germinating cyst stage compared with that at the mycelial stage. As noted by Judelson et al. [43], following the examination of the *P. infestans* transcriptome, genes were upregulated exactly before their products are required; hence, the upregulation of elicitin-like genes during these stages strongly suggests a role in the pre-infection and early infection stages and further supports their function as a pathogenicity factor. Similarly, transcriptome studies of other *Phytophthora* species have defined the differences in the spatial and temporal expression of the *ELI* and *ELL* genes [44,45]. Based on these and other studies, further analyses using transcriptomics within a specific, preferably model host, will provide new information about the roles of both elicitins and elicitin-like proteins.

#### 5.1.3. Proteomic Approaches

The study of whole-organism proteomes using high-throughput proteomics approaches is now the method of choice for an understanding of pathogenicity and virulence-associated proteins. [46]. Additionally, phyto-pathoproteomics [47], the study of the proteome of the pathogen, host and their interaction can be explored using these proteome tools. For example, over a hundred pathogenicity and virulence-related factors of the non-oomycete *Fusarium graminearum* were discovered with the use of NGS and proteomics [48]. Another efficient and accurate tool is iTRAQ (isobaric tags for relative and absolute quantification) [49,50] for the analysis of protein profiles and signaling pathways. In *N. benthamiana*, this approach led to the identification of 2964 proteins in plants treated with the elicitin INF-1, two of which were shown to be essential in INF-1-triggered cell death responses. Further use of the iTRAQ method will allow the elucidation of the molecular mechanisms that underlie elicitin-triggered immunity [51]. A study using another proteome tool, SWATH-MS (Sequential Window Acquisition of All Theoretical Mass Spectra) [52], revealed the differential expression of 80 proteins in *Q. suber* infected with *P. cinnamomi* that provided insights into the association between pathogen invasion and the subsequent plant response. Another technique that holds prospects for the identification of elicitin functions and proteome relationships is shotgun proteomics [53], which has been used, for example, to detect over 500 different peptides in culture filtrates of the mycorrhizal fungus *Rhizophagus irregularis*, and which has greatly expanded our knowledge of proteins that are involved in the establishment of symbiosis [54].

#### 5.1.4. Metabolomics and Cell Signaling

The intermediates and products of biochemical pathways are metabolites, making the study of them pivotal for the corroboration of genomic, proteomic and transcriptomic data. Through the use of MS and nuclear magnetic resonance (NMR) spectroscopy, metabolomics has emerged as a powerful tool to monitor the myriad of molecules that are produced during plant–pathogen interactions [55,56]. Elicitins have been reported to stimulate changes in the concentration of ROS and calcium ions, messengers that play an intrinsic role in the control of a host plant’s cellular processes and development, hormone production and immune response [57]. In *Nicotiana*, for example, alongside the induction of HR, the elicitin treatment led to the activation of both the jasmonic acid (JA) and salicylic acid (SA) biosynthesis pathways. The knowledge of such metabolites that elicitins specifically induce at both the cellular and subcellular levels will enable us to use the metabolites as molecular markers to further elucidate the involvement of elicitins in the generation of these and other complex signaling networks that lead to either plant susceptibility or resistance.

### 5.2. Gene Editing Using CRISPR/Cas

The use of CRISPR/Cas has provided unprecedented advancements in gene editing and has the potential to revolutionize our approach to disease suppression and crop improvement [58]. Genome engineering of oomycetes has been limited due to the extremely low rate of success with homologous recombination, making the study of targeted gene mutations and gene replacements challenging. As such, the study of elicitin knockdown transformants of *Phytophthora* has been difficult, mainly due to the recalcitrant nature of this genus to transformation [59]. In 2017, Fang et al. [59] developed a protocol for gene editing of *Phytophthora sojae* using a CRISPR/Cas system that enabled heritable genome modifications. The success of this technique signified that the protocol could be used for most culturable oomycetes and, hence, opened up a powerful new research capability for the use of gene editing for the identification of the role of elicitins in pathogenicity.

**Figure 5:**
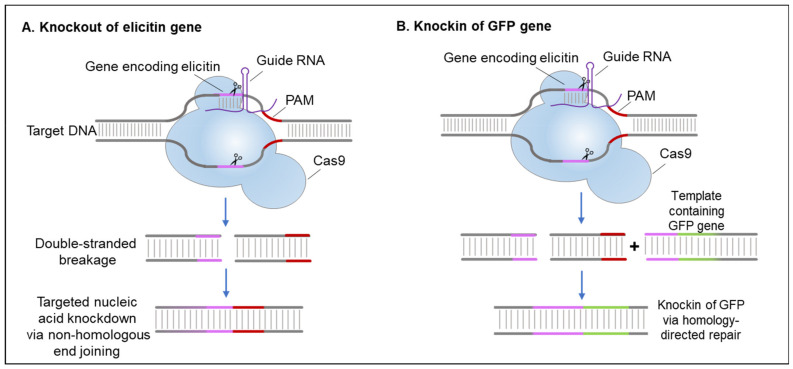
Concept of using site-specific CRISPR/Cas9-mediated gene editing to understand the functions of elicitins. (**A**) The generation of elicitin gene mutants with targeted nucleic acid knockdown to monitor the differences in infectivity of the pathogen. Knockout of the whole elicitin gene may lead to the inability of the pathogen to acquire sterols. (**B**) Knockin of the green fluorescent protein (GFP) (or similar markers) at the elicitin gene locus through homologous recombination to produce labeled elicitin proteins for detailed subcellular visualization and monitoring. Pink: elicitin gene, red: protospacer adjacent motif (PAM), purple: guide RNA and green: gene for the fluorescent tag.

The result of targeted gene mutation using this technique was elegantly shown for the 15-kDa glycoprotein Ppal15 kDa, which was found abundantly in culture filtrates of *P. palmivora* [60], where a significant role of the protein in infection structure development and pathogenicity was demonstrated. Furthermore, in one of the first studies to use this technique in oomycetes, Fang et al. [61] used CRISPR/Cas9 to disrupt and replace effector genes in *P. sojae*, demonstrating the power of this tool for the study of functional genomics in *Phytophthora*. In Figure 5, we present a conceptual overview of the use of CRISPR/Cas9 for the knockout and knockin of elicitin genes and couple that with the ability to visualize editing with, for example, a GFP reporter that can be quite quickly used to further enhance our current understanding of the function and location of elicitins during plant infections.

Previously termed a ‘primary weapon’ to ensure disease progression, CRISPR/Cas9 can now be used to monitor the pathogenicity, for example, of *Phytophthora* spp. in the absence, or mutation, of an elicitin gene. GFP is widely used as a marker for in vivo and in vitro studies due to their ease of expression, even in the absence of any exogenous substrate or cofactor [62]. Thus, coupling of the endogenous elicitin gene with GFP through homologous gene recombination techniques ensures targeted editing and has provided valuable insights in many studies [63]. While most reports have shown negligible effects of GFP tagging on cellular functions, studies would need to show that the resulting GFP or similar marker-tagged elicitin protein would not impair or alter the protein function.

The application of CRISPR/Cas has addressed many of the previous obstacles for editing oomycete genes, and successful transformants have been generated, such as from the use of a CRISPR/Cas12a system in *P. infestans*, which has enabled a greater capability for gene editing in oomycetes generally [64]. Similarly, point mutations introduced into *P. sojae* [65] using CRISPR/Cas9 conferred resistance to the fungicide oxathiapiprolin. Muñoz et al. [66] outlined the potential use of CRISPR/Cas to produce nonvirulent mutant strains of filamentous fungal pathogens such as *Fusarium* spp. and then, through subsequent competition with the wildtype proposed to reduce occurrence of the disease. Even though much of the research to date has been proof-of-concept, the use of CRISPR/Cas for the targeted modification of a pathogen should open up possibilities to control plant diseases. However, we cannot underestimate the challenge of translating the results of laboratory studies on oomycetes, for example, into applications in the field [67]. We need to develop greater knowledge about genetic stability, especially in oomycetes, and, also, whether the approach of using CRISPR/Cas is feasible and cost-effective in the field. Hence, in-line with the focus of our review, and similarly to Paul et al. [68], we see the way forward being the use of not only CRISPR/Cas but, more broadly, synthetic biology techniques to dissect and understand various pathosystems, including those involving oomycetes, with the aim of the diversification of applications with time.

## 6. Association of Elicitins with Other Organisms

Even though this review has focused on elicitin-producing oomycetes that cause plant disease, it is worth considering oomycetes that may utilize elicitins to promote diseases in organisms other than plants. Most of the pathogenic oomycetes only infect plants; however, *Pythium insidiosum* infects both plants and mammals. Identified as a notorious oomycete, *Py. insidiosum* causes a rare, life-threatening infectious disease, pythiosis, in humans and animals such as dogs and horses. A number of studies [69,70,71] have shown that pythiosis commonly occurs in tropical and subtropical areas, with cases reported from various countries, including Thailand, Australia and Malaysia. Human pythiosis was recorded by Krajaejun et al. [71] as causing 29% mortality and increasing to 40% when it reaches the vascular tissues. The elicitin-like glycoprotein gene *ELI025* of *Py. insidiosum* has an unknown function but is highly expressed and upregulated at human body temperature. Even though BLAST analyses of the ELI025 protein showed a surprisingly high similarity with other oomycete elicitins, the exclusiveness of this elicitin to *Py. insidiosum* has been exploited for clinical applications [19]. Thus, an anti-ELI025 antibody-based immunohistochemical assay was established to diagnose pythiosis and showed 100% detection sensitivity and accuracy [72].

Despite the significant yet unresolved involvement of elicitins in pathogenicity, we cannot overlook the fact that there is a lack of understanding of their functionality in other non-plant hosts. There is a need for studies on elicitins in other oomycetes that cause diseases in animals, such as white spot disease caused by *Aphanomyces invadans* in fish [73]. A further example is *Py. guiyangense,* an oomycete used as a biocontrol agent against mosquitoes, whereby an upregulation of elicitin genes [74] was observed during the mosquito larvae infection stage. Clearly, these non-plant interactions that involve elicitins require deeper investigation using the approaches described here, and they may shed further light on the interactions of elicitins with plants.

## 7. Conclusions

Elicitins were first described in the 1980s; however, it is only in the last few years that the functions of these cryptic proteins can now be elucidated by using, among other approaches, gene editing, bioinformatics and multi-omics approaches. Even though bioinformatic tools and, indeed, CRISPR/Cas have been available for some time, they have not been applied to an understanding of the biology of elicitins and their role in plant–pathogen interactions. Now is the time to utilize these approaches. We highlighted how an analysis of these important proteins using these new and more powerful techniques may provide a deeper understanding of the role of elicitins in plant–pathogen interactions. Two striking questions remain that need to be addressed using the approaches outlined here. Firstly, what enables an elicitin to function as either a virulence or avirulence factor, and how is that dependent on the plant host species? Secondly, will specifically targeting elicitins enable us to attenuate the disease-causing ability of pathogenic oomycetes? The answers to these questions will provide another tool for the protection of crop plants in particular and the development of novel control strategies more broadly for oomycete pathogens, for which there are currently few effective and long-term controls.

## Data Availability

All figures in this manuscript are original.
